# Evidencing the importance of the functional unit in comparative life cycle assessment of organic berry crops

**DOI:** 10.1007/s11356-024-32540-6

**Published:** 2024-02-24

**Authors:** Reina Pérez, Fernando Argüelles, Amanda Laca, Adriana Laca

**Affiliations:** Department of Chemical and Environmental Engineering, University of Oviedo, C/ Julián Clavería S/N, 33006 Oviedo, Asturias Spain

**Keywords:** FU, NDU, Farm-gate price, Environmental impacts, Berry production, Carbon footprint, LCA

## Abstract

LCA methodology provides the best framework to evaluate environmental impacts in agriculture systems. However, the interpretation of LCA results, in particular when the objective was to compare different production systems, could be affected by the selection of the functional unit (FU). That is why an accurate definition of the FU, in agreement with the function considered for the systems analysed, is essential. In this work, the organic production at small scale of blueberry, raspberry, blackberry and cape gooseberry in North Spain has been analysed following LCA methodology. Although a different distribution of environmental loads was obtained for each crop, in all cases, the main contributions to most of the considered environmental categories were electric and fertiliser consumptions. The different production systems have been compared on the basis of the environmental impacts associated considering different FUs, i.e. based on fruit mass, cultivated area, farm-gate price and nutritional quality of fruits. Carbon footprints (CF) have been also calculated. It was observed that the order of the crops with respect to their environmental performances was the same for the blueberry and raspberry crops (with the lowest and the highest CF, respectively), independently of the selected FU, whereas the order of the blackberry and cape gooseberry crops was interchanged, depending on the FU used. This work supports the need of being aware of the final objective of the orchards when choosing the FU (i.e. producing fruits, cultivating an area, economic benefits or nourishing people), so that valid conclusions can be achieved from the environmental comparison, even for different agricultural products.

**Supplementary Information:**

The online version contains supplementary material available at 10.1007/s11356-024-32540-6.

## Introduction

According to the European Commission, life cycle assessment (LCA) provides the best framework to evaluate the environmental impacts of a production system, and the European Union has created an initiative to harmonize this internationally standardised methodology (European Commission 2005). LCA is an interesting environmental tool that can be used to improve material and resource management, reduce wastes, optimise production steps and evaluate new products or technologies, among others. The ongoing challenges in LCA are mainly related to the interpretation and comparison of results and the unification of criteria in different sectors and activities. Several studies have been carried out to adapt this methodology to the agricultural sector (Nitschelm et al. 2021; Tragnone et al. 2022), so that, nowadays, its use is well established in this research area for evaluating environmental impacts (Soulé et al. 2021).

LCA studies allow the identification of opportunities to obtain sustainable high-yield food production systems, including organic systems. Specifically, LCA analysis has shown that vegetables and fruits entail lower greenhouse gas (GHG) emissions per kg of product compared with other foodstuffs (Mogensen et al. 2009). Recently, different works have focused on comparing conventional vs organic crops in terms of sustainability, in an attempt to minimize environmental impacts derived from food production (Cucurachi et al. 2019) which is a key aspect regarding the UN 2030 Agenda for Sustainable Development (UN 2022).

According to ISO 14040 and 14044 standards, four phases have to be included in an LCA: goal and scope definition, inventory analysis, impact assessment and interpretation. The quality of the results of an LCA depends on the robustness, the reproducibility and the reliability of the procedures carried out during these four steps (Bongono et al. 2020). In this regard, the functional unit (FU) is a key element of LCA, which has to be clearly defined in relation with the objectives of the study. ISO 14040 defined functional unit as the *quantified performance of a product system for use as a reference unit*, and this standard, as well as ISO 14044, highlights the FU importance to provide reliable LCA results in comparative assessments. FU refers to calculation basis on which resources and emission balances have to be made, and it is the reference to which all other data in the assessment are normalised (Weidema et al. 2004).

A clear functional unit definition that is supported by the scope of the study is essential in performing an assessment, since inconsistency in system boundaries and functional units can lead to miscommunication of results and may affect interpretation of environmental impacts (DeMarco and Fortier 2022). However, FU selection is usually arbitrary depending on the sector and case of study and a high part of subjectivity is let to the LCA practitioner. In general terms, FU should be quantifiable, include units and consider temporal coverage (Matthews et al. 2014). In case of multifunctional systems, such as agricultural sector, many difficulties persist for the selection of FU. According to Matthews et al. (2014), FU should include several dimensions which are the responses to “What?”, “How much?”, “How well?”, “For how long/how many times?” and “Where?” of the object of study. Difficulties of the selection of FU lie in the need to determine and prioritize the functions of the system and decide quantitative or qualitative data, taking in consideration the end use of the product and stakeholders, and the avoidance of generalization. In conclusion, it should be closest to the end use and should be aligned with the objective of the study, i.e. substitution of a product, comparison of products… (Bongono et al. 2020; Weidema et al. 2004). Hence, it is obvious that the choice of the FU is critical and could significantly influence the conclusions of the study (van der Giesen et al. 2020).

An important question to answer at this respect is what could be the best functional unit in different systems, and, specifically, regarding the present work, in agriculture systems. Generally, agricultural LCA studies calculate environmental impacts based on mass (Fotia et al. 2021; Laca et al. 2020; Ronga et al. 2019). Additionally, eco-efficiency of a particular crop could be quantified by means of several indicators expressed per weight of the raw material or product (e.g. kg or ton). But if an agricultural study is focused on the land use of a region or on production intensity, the functional unit should be based on surface units (e.g. ha or m^2^) (da Silva et al. 2014). Moreover, using a mass-based functional unit, which is predominant in current life cycle assessment practice, despises of the negative environmental consequences of agricultural system intensification (Salou et al. 2017). In addition, organic farming may have higher impacts per kilogram compared to conventional systems, mainly due to their lower crop yields that implies the use of bigger land areas to produce the same amount of food. In contrast, organic crops have lower than conventional systems when impacts per ha are considered (Cucurachi et al. 2019). For LCA studies of agricultural products, the use of both mass- and area-based FUs is advised. This recommendation becomes very important when orchards with different yield are compared, e.g. conventional versus organic systems (Salou et al. 2017).

Recent studies tend to include economic perspective to consider the sustainability of a product. Da Silva (2014) explored the use of the economic value at the farm gate as an alternative FU. This author determined that the FU, which strongly depends on the viewpoint considered, is a key element to compare systems. In addition, consumers are giving great importance to nutritional quality.

Therefore, new lines of research have implemented a unique functional unit based on the nutritional value of a food product with the aim of supporting sustainable decisions. Several approaches have been carried out to identify a suitable method for nutrient evaluation, without reaching a consensus so far. Sonesson et al. (2019) defined two nutrient methods, a nutrient quality index (NQI) and a nutrient rich foods index 9.3 (NRF9.3), which consider the content of qualifying nutrients (i.e. nutrients positive for health as protein, fibre, vitamins A, C and E, calcium) and disqualifying nutrients (i.e. nutrients whose intakes should be limited as saturated fat, sodium, and added sugar). Dooren (2016) proposed a Nutrient Density Unit (NDU) that reflects the food’s ‘function’ of supplying the essential macronutrients according to human metabolic energy needs. Masset et al. (2015) created his own sustainability score, defining a sustainable food product as that which meets three criteria: low environmental impact, high nutritional quality and affordable price.

The goal of the present study was to investigate the influence of the FU choice on the comparison of different crops from an LCA perspective. In particular, for first time blueberries, blackberries, raspberries and gooseberries organic productions at small scale were compared using four different functional units (1 kg of fruit, 1 ha of land, 1 euro of farm-gate price, 1 NDU). In addition, carbon footprints of the different berries have been obtained in each case, analysing the effect of FU on the gathered conclusions.

## Materials and methods

### System description

This study was carried out in the Principality of Asturias, a region in Northern Spain, characterised by an oceanic climate. This rainy and moderately warm climate provides excellent conditions for fruit and vegetable production, especially for berries crops, such as blueberries, blackberries and raspberries, including also other less popular berries, such as gooseberries. In general, the size of Asturias berry crops is smaller than the average sizes usually found in other European countries.

Crops included in this study share the following characteristics: they are young, family owned and sited below 250 m of altitude with cultivated areas of less than 10,000 m^2^. All the considered systems have ecological certification based on specific principles, which comply with organic production standards established by Regulation (EU) 2018/848 on organic production and labelling of organic products (Commission 2018). Table 1 summarises the principal characteristics of each system analysed in this study.

**Table 1:** Overview of the main characteristics of the production systems studied

	Blueberry	Raspberry	Blackberry	Gooseberry
Organic certification	Yes	Yes	Yes	Yes
Location*	Tapia de Casariego	Siero	Siero	Ribadesella
Altitude (m)	40	250	250	125
Occupation crop (ha)	0.810	0.003	0.002	0.500
Type of land	Meadow	Meadow	Meadow	Meadow
Type of soil	Sandy loam	Clay loam	Clay loam	Clay loam
Cultivated varieties	*Vaccinium myrtillus* L	*Rubus idaeus* L	*Rubus* sp.	*Physalis peruviana* L
Age of plants (years)	*7*	*7*	*7*	*2*
Number of plants	3,012	50	10	1,000
Fruit production (kg/year)	4,990	25	62	800
Land productivity (kg/ha*year)	6,160	8,333	31,000	1,600
Sale system	Direct	Direct	Direct	Direct
Irrigation water origin	River	Rain	Rain	Creek
Type of fertiliser	Ecological	Ecological	Ecological	Ecological
Fertilisers consumption (kg/year)	109.48	13.30	8.69	202
Waste treatment of pruning	composting	composting	composting	composting
Pruning waste (kg/year)	3750	2.5	2.50	200
Brushcutting waste (kg/year)	400	3.0	2.00	100

^*^All sited in Principality of Asturias (Spain)

Blueberry orchard was located on the Western of Asturias, whereas raspberry and blackberry orchards belong to the same farm located in the centre of the region. The cultivation of these two berries was carried out in different adjacent smallholdings, perfectly delimited. Cape gooseberry orchard was located in the West, near to the Asturian coast.

Blueberry and cape gooseberry systems had an extension of 0.8 and 0.5 ha respectively, in sharp contrast with raspberry and blackberry orchards that occupied only 0.003 and 0.002 ha, respectively. The blueberry had the highest production, with 4990 kg of fruit per year, followed by cape gooseberry with 800 kg per year, whereas the production of raspberry and blackberry was notably lower with 25 and 62 kg per year, respectively.

Data considered in this study corresponds to the year 2020 in all cases.

### Life cycle assessment

#### Objective, functional unit and boundaries

In this study, LCA methodology has been employed in order to compare the environmental impacts associated to the organic production of different berries. Different functional units have been tested in order to know if their choice may significantly affect the LCA results and the conclusions that can be extracted. Specifically, four different functional units have been included:

1. Mass-based FU
  One kilogram of fruit produced was defined as mass-based FU. The objective was to obtain a certain quality of any of the berries considered at the farm gate. This FU is related with the production efficiency of the farm.
2. Area-based FU
  One hectare of land occupied by the orchard during a year was defined as area-based FU. The land occupation included farm land for crops and buildings related. The objective was to cultivate a certain area of land with any of the berries considered.
3. Price-based FU
  The amount of any of the fruits sold by 1 euro at the farm gate was considered as FU. Only the economic value of the products at the farm-gate is included, excluding transport or delivery costs. It was taken as a reference of the prices at origin for blueberries and raspberries in year 2020 from the Ministry of Agriculture, Livestock, Fisheries and Sustainable Development of the Andalucía Government (CAGPDS 2020a, b). Gooseberries and blackberries’ farm-gate price was obtained by extrapolation between average sale price to the final consumer and benefits’ ratio (calculated based on gate and sale prices of blueberries and raspberries). Therefore, the farm-gate prices considered were 4.06, 5.37, 8.27 and 6.44 euros per kg of blueberry, raspberry, blackberry and cape gooseberry, respectively. The objective in this case was to produce the amount of any of the berries that can be sold at a certain price.
4. Nutritional-based FU
  As FU, it was taken 1 Nutritional Density Unit (NDU). This FU allows the analysis of the environmental impacts of a product in relation to its nutritional function (Dooren 2016). It takes in consideration individual macro- and micronutrients related to energy density. The mass of fruit equivalent to 1 NDU was calculated from the nutritional information of each fruit. NDU values obtained were 16.07, 43.13, 43.30 and 20.08 NDU per kg of blueberry, raspberry, blackberry and cape gooseberry, respectively (Jaiswal 2020; Ponder and Hallmann 2020; Ramadan 2011; Simmonds and Preedy 2015). The objective in this case was to produce the amount of any of the berries considered that can supply certain amount of nutrients when it is consumed.

Regarding the boundaries, this study included from the extraction of materials to the orchard gate (“*cradle to gate*”). Practices considered in this study were as follows: manufacturing processes for inputs, transport of fruits to storage place, packaging, emissions to soil and atmosphere and waste management. Raw material transport and distribution to points of sale have not been taken into account. Data from crops in productive stage during 1 year were considered for the analysis, excluding nursery, establishment, low production years and dismantling. Similarly, the construction of infrastructures, buildings and other facilities existing in the crop were not considered either. Likewise, inputs/outputs that amounted for less than 1%, such as some minor fertiliser ingredients, were not included. According to PAS 2050 (British Standards Institution 2007), CO_2_ uptake was included in LCA because more than 50% of the mass of biogenic carbon remained retained on the orchards for 1 year or more (the four berries considered are small to medium-sized perennial woody plants).

In case of blackberry and raspberry, it was necessary to allocate some of the inputs/outputs of the system among the fruits cultivated in the farm. Specifically, allocation was area-based to calculate tap water, electricity, fossil fuel and fertiliser consumption, whereas packaging materials were considered proportional to the mass of each berry produced.

#### Inventory analysis

Inventory net data of berries orchards are organised in the subsystems shown in bold letter in Table 2. Principal inputs were land use, CO_2_ uptake and consumptions (water, electricity, fossil fuel, plastic, paper, mulching and fertilisers). The main outputs of the orchard were wastewater (to treatment), solid waste (to landfill), plastic and paper wastes (to recycling), emissions to air from fossil fuel, fertiliser and composting and emissions to soil from fertilisers. Data were obtained through detailed questionnaires, personal interviews with farmers, visits to the facilities and/or from reliable literature sources. The blueberry crop used an automatic irrigation system for watering and fertilisation, whereas the other producers carried out these tasks manually. Electricity was supplied by an external company in all cases.

**Table 2:** Inventory data of the systems analysed

Principal net inputs of the systems					
Subsystem	Blueberry	Raspberry	Blackberry	Gooseberry	UNITS
Land use	8,100	30	20	5000	m^2^
Water consumption					
Tap water	16.00	1.55	1.03	0.01	m^3^
Water natural origin	813.39	0.62	0.12	1.00	m^3^
Electric consumption					
Electricity company	1,136	377	251	1228	kW*h
Fossil fuels consumption					
Gasoline for machinery	0.20	0.01	0.007	-	m^3^
Diesel for transport	-	-	-	0.02	m^3^
CO_2_ uptake	60,852.11	234.51	7.90	239.10	kg
Plastic consumption					
RPET plastic	7.20	0.37	1.05	-	kg
PP plastic	-	-	-	6.00	kg
PVC plastic	-	-	-	0.55	kg
PS plastic	-	-	-	3.00	kg
Paper consumption					
Kraft paper	-	-	-	20.00	kg
Cardboard	-	-	-	25.00	kg
Mulch (straw)	-	37.50	7.50	-	kg
Substratum	-	-	-	15.00	kg
Fertilisers consumption					
Total nitrogen (N)	0.98	0.51	0.34	61.20	kg
Water soluble potassium oxide (K_2_O)	3.40	0.60	0.40	-	kg
Water soluble potassium (K)	11.60	-	-	-	kg
Potassium carbonate (K_2_CO_3_)	20.46	-	-	-	kg
Sulphur trioxide (SO_3_) soluble in water	-	0.15	0.10	30.00	kg
Total organic matter (TOC)	-	5.91	3.94	-	kg
Phosphorous pentoxide (P_2_O_5_)	-	0.29	0.19	-	kg
Calcium (Ca)	2.87	0.47	0.31	-	kg
Amino acids	5.45	-	-	1.10	kg
Humic and fulvic acids	25.50	3.81	2.54	0.20	kg
Magnesium (Mg)	-	0.21	0.13	2.40	kg
Principal net outputs of the systems					
Subsystem	Blueberry	Raspberry	Blackberry	Gooseberry	UNITS
Waste water	16.00	1.55	1.03	-	m^3^
Fossil fuel emissions to air					
Carbon dioxide (CO_2_)	455.36	23.09	15.39	186.42	kg
Methane (CH_4_)	0.22	0.01	0.007	0.01	kg
Dinitrogen monoxide (N_2_O)	0.02	0.001	0.00	0.01	kg
Carbon monoxide (CO)	13.68	0.70	0.46	0.44	kg
Hydrocarbons (HC)	2.24	0.11	0.07	0.82	kg
Nitrogen oxides (NO_x_)	2.11	0.11	0.07	2.43	kg
PM	-	-	-	0.00	
Sulphur oxides (SO_x_)	-	-	-	0.68	kg
Nitrogen (N_2_)	1142.99	57.96	38.64	662.36	kg
Water (H_2_O)	95.21	4.82	3.21	69.91	kg
Oxygen (O_2_)	12.88	0.65	0.43	56.49	kg
Fertiliser and composting emissions to soil					
Total nitrogen (N)	0.20	0.10	0.07	12.24	kg
Phosphorous pentoxide (P_2_O_5_)	-	0.06	0.03	-	kg
Calcium	11.43	0.09	0.06	-	kg
Potassium	0.56	0.05	0.03	-	kg
Sulphur (SO_2_)	-	0.02	0.01	4.80	kg
Total organic matter (TOC)	80.00	12.72	12.20	110.03	kg
Magnesium (Mg)	-	0.04	0.02	0.53	kg
Fertiliser emissions to air					
Ammonia (NH_3_)	0.09	0.04	0.03	5.51	kg
Nitrogen dioxide (NO_2_)	0.01	0.005	0.003	0.60	kg
Dinitrogen monoxide (N_2_O)	0.02	0.005	0.003	0.55	kg
Composting emissions to air					
Methane (CH_4_)	16.60	0.17	0.05	2.20	kg
Dinitrogen monoxide (N_2_O)	0.99	0.01	0.002	0.13	kg
Ammonia (NH_3_)	0.99	0.01	0.002	0.13	kg
Solid wastes					
Waste to landfill	0.00	0.82	2.04	2.00	kg
Plastic wastes					
HDPE plastic to recycling	3.04	0.00	0.00	0.00	kg
Paper wastes					
Paper waste to recycling	-	-	-	1.05	kg
Products	4990	25	62	800	kg

Fertilisers were included in the analysis by means of calculating active ingredients of each product (Mohamad et al. 2014). Emissions to land derived from fertilisation have been included considering that 20% of the applied product leachates to the soil (INIA 2013). Emissions from fertilisers to the atmosphere were estimated following the methods proposed by the Intergovernmental Panel on Climate Change (IPCC) and the Ministry of Agriculture, Fisheries and Food (MAPA) of Spain and using emission factors found in literature (Aalde et al. 2006; Doorn et al. 2006; Lasco et al. 2006). Specifically, the factors employed were 0.09 g NH_3_, 0.01 g NO_2_ and 0.009 g N_2_O per g of nitrogen contained in the fertiliser applied to the soil.

In all cases, heavy machinery was not employed in orchards, and fruit harvesting and tree pruning were carried out manually. Emissions derived from fossil fuels consumed on clearing processes and transport within the farm were included considering IPCC and national inventory techniques (MITECO 2012, 2019; Waldron et al. 2006) and according to Reşitoglu and Altinişik (2015).

Compost emissions derived from the in situ decomposition of organic wastes were calculated considering that 1 kg of wet treated waste emits 4 g CH_4_, 0.24 g of NO_2_ and 0.24 g of NH_3_ (Doorn et al. 2006; MITECO 2012).

The CO_2_ uptake was calculated considering net photosynthetic rate for each berries in 1 year (Casierra-Posada et al. 2008; Casierra and L. Hernández 2006; Enciso and Gómez 2004; Funk 2008; Jara-Peña et al. 2003; López-Sandoval et al. 2018; Mesa Torres 2015; Nemeth et al. 2017; Vico et al. 2018).

Some more details about the boundaries and inventory analysis can be found in Perez et al. (2022).

#### Impact assessment

ReCiPe 2016 Midpoint (H) V1.1 method and Ecoinvent 3.4 database have been employed for the quantification of environmental impacts derived from the different production systems by means of Simapro 9.5.0.0 software (Pré-Consultants 2010). ReCiPe method includes 18 categories of environmental impact, i.e. global warming (GW), stratospheric ozone depletion (SOD), ionizing radiation (IR), ozone formation human health (OFHH), fine particular matter formation (FPMF), ozone formation terrestrial ecosystems (OFTE), terrestrial acidification (TA), freshwater eutrophication (FE), marine eutrophication (ME), terrestrial ecotoxicity (TEC), freshwater ecotoxicity (FEC), marine ecotoxicity (MEC), human carcinogenic toxicity (HCT), human non-carcinogenic toxicity (HNCT), land use (LUC), mineral resource scarcity (MRS), fossil resource scarcity (FRS) and water consumption (WC) (Huijbregts et al. 2016).

### Carbon footprint (CF)

Greenhouse Protocol (GHG) V.1.03 has been used to obtain the carbon footprint (kg CO_2_eq per functional unit) of the four berries analysed for the four FUs considered. Ecoinvent 3.4 database and Simapro 9.5.0.0 software (Pré-Consultants 2010) were employed. CF values were obtained considering fossil and biogenic CO_2_, according to ISO 14067 (ISO 2018).

## Results and discussion

### Crops and main contributions to the impacts derived from berry production

Table 2 shows a detailed inventory of the principal inputs and outputs of the systems analysed. As the values given are the raw data corresponding to 1 year, great differences can be observed, depending on the diversity of sizes in the studied orchards. Raspberry and blackberry crops together used only 50 m^2^ of soil, far away of the 8100 m^2^ and 5000 m^2^ used by blueberry and cape gooseberry crops, respectively. The first two cases belong to the same multi-product farm. Multi-cropping systems are quite common for European small farms with organic production.

As shown in Table 1, productivity (berries per land) was independent on the size of the orchard, being the lowest for the cape gooseberry with 1600 kg/ha year and the highest for the blackberry with 31,000 kg/ha year. McEachern et al. (1997) indicated that blackberry plants may produce for 15 years if they are accurately managed, and the best production is usually from the third to the eighth year; therefore, the blackberry crop studied here is in its optimum age for production (7 years). With respect to the analysed organic cape gooseberry crop, Parker (2012) disclosed that *Physalis peruviana* yield achieves its maximum in the second year and drops in the following years if the crop is maintained for that long. In the case study analysed here, and according to the previous author, the plants were in their optimum age (2 years); however, the productivity obtained here was much lower than the 14,000 kg/ha achieved in Colombia, main producer of this berry. This can be due to different reasons, for example the different climatology. Besides, Quevedo García et al. (2015) reported that plant density and training system are determinant to optimise the yields of this crop. In addition, it is necessary to consider that, in general, organic agriculture emits less pollutants per unit of occupied land, but higher per unit of product due to its lower yields per unit of area (Basset-Mens and Van Der Werf 2005).

Raspberry and blueberry productivities were in the medium with productivities of 8330 and 6160 kg/ha year, respectively (Table 1). These values are slightly higher than the average productivities given in the Spanish Statistics for Organic Production (MAPA 2021), i.e. 6100 kg/ha for raspberry and 4300 kg/ha for other berries (different from strawberry and redcurrant). It is necessary to take into account that productivities can significantly vary depending on the location, climate conditions, agricultural practices and orchard age. As other orchards, raspberry yield is affected by the plantation age, and full crops normally are attained by the third growing season (Perasović 2013; Wróblewska et al. 2020). In the evaluated crop, the age of the raspberry trees was 7 years, so the orchard has overcome this stage. The North of Spain, in particular Asturias, provides excellent conditions for blueberry and blackberry crops with average organic regional productivities for berries (different from strawberry, raspberry and redcurrant) around 10,000 kg/ha in 2020 (MAPA 2021). Specifically, it should be considered that blueberry plants usually require 6 to 8 years to reach full production; accordingly, the studied orchard is in the optimum age (7 years) (Oregon State University 2023). In this sense, Salvo et al. (2011) found that the number of fruits per bud depends significantly on the variety and age of the plant. In addition, Palma et al. (2023) indicated that the number of flower buds per cane showed a positive relationship with cane diameter and cane age. These authors also claimed that as blueberry bushes need regular pruning to establish and maintain a balance between the vegetative and reproductive parts of the plant, pruning practices are determinant on fruit yield and quality.

Another aspect to be noted is the high water demand of the blueberry crop, with an annual water consumption of 813 m^3^ per year (in addition to the raining water), which translates into 1023 m^3^/ha. This orchard had an irrigation system consisting on a network of PVC pipes underground, and fertiliser was supplied in the irrigation water. Hose irrigation and cover fertilisation were the usual practices in the rest of the crops. Electricity consumed per kilogram of fruit was quite higher for the smallest orchards (15 and 4 kWh/kg for raspberry and blackberry, respectively, vs 0.2 and 1.5 kWh/kg for blueberry and cape gooseberry, respectively). Besides, an uneven consumption of fertilisers could be observed among the crops. It is noteworthy that the farm that presented most plants (blueberry) had the lowest fertiliser consumption, 0.036 kg per plant and year, compared to the 0.20–0.90 kg/plant of the rest of the berries. Composting emissions to air and soil came from the in situ decomposition of vegetal wastes removed during street cleaning and pruning, which differed substantially depending on the type of crop, i.e. between 600 kg/ha (cape gooseberry) and 5123 kg/ha (blueberry).

LCA characterization results (Fig. S1, S2, S3 and S4) indicated that the most impacting subsystem was, in all cases, electricity consumption, which affects specially IR and FE with contributions higher than 70%. This strong contribution of the electricity subsystem is especially important for the raspberry and blackberry orchards, where this contribution is higher than 60% in 13 of the 18 considered categories. This is due to the high specific electricity consumption, above commented, of this small farm. Another important subsystem was fertiliser consumption, which contributed to environmental impacts in all crops and more notably in the cape gooseberry orchard, with contributions higher than 50% in the TEC and MRS categories. The emissions to air from fertiliser and composting significantly contributed to SOD category in all cases. In addition, fossil fuel consumption also had important contributions in all the systems, in particular in the FRS category. It is remarkable that mulch used to protect soil had in the case of raspberry a contribution higher than 10% in the ME, LUC and WC categories, due to the use of a high amount of straw mulch (1.50 kg/kg of fruit). This type of mulch material is frequently used in organic crops, especially for berries, watering it regularly to avoid straw being blown away by the wind. As expected, land use, water consumption and wastewater treatment affected mainly the LUC, WC and ME categories, respectively. For blueberry and raspberry crops, the harmful effects on GW category were overcompensated by the CO_2_ uptake due to the growth of plants and fruits.

### Environmental comparison of berries production systems

#### Mass-based FU

**Fig. 1:**
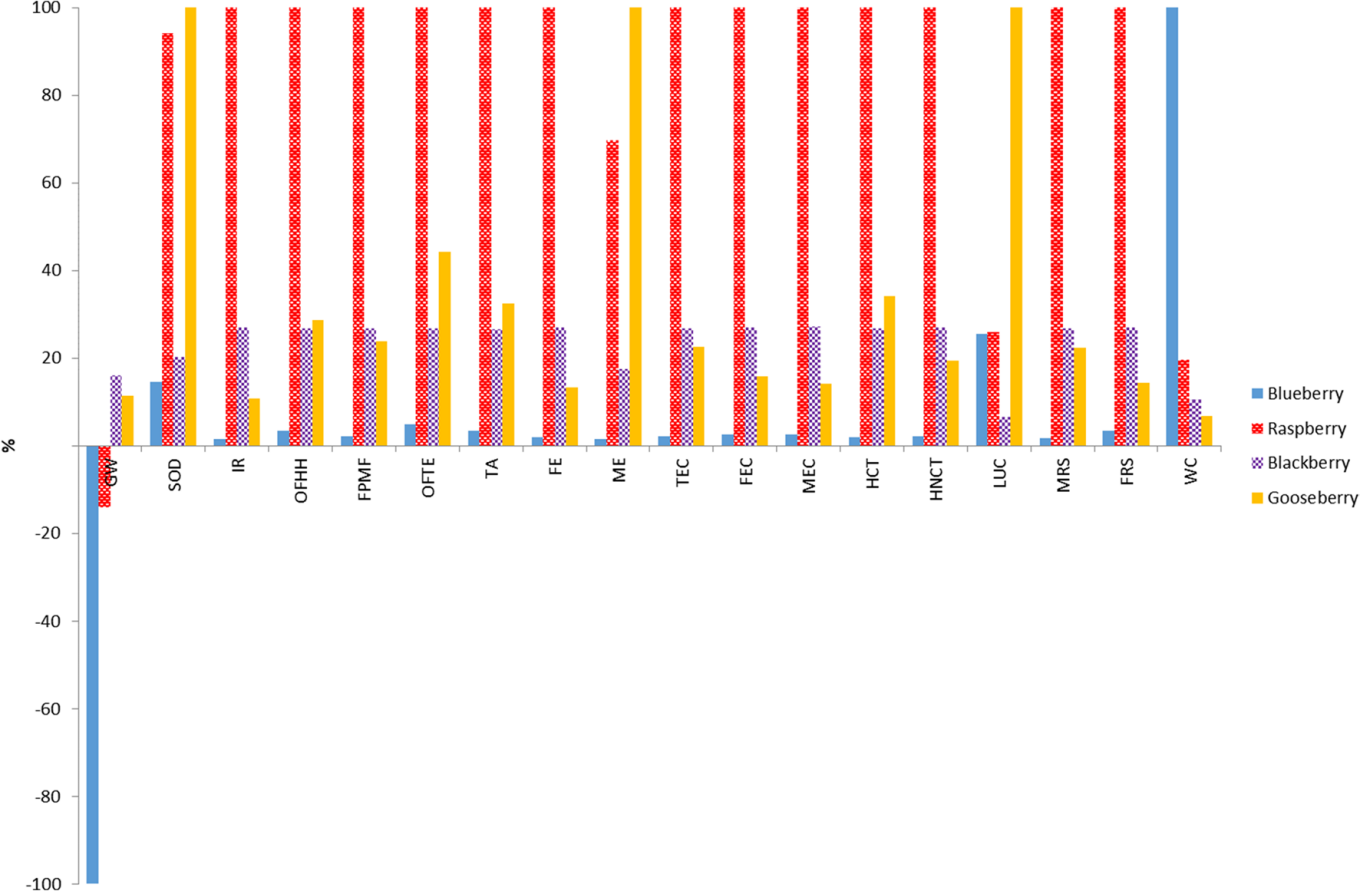
Comparison of characterization results obtained for different systems using Recipe Midpoint (H) method (FU: 1 kg fruit)

In Fig. 1, results of LCA using a mass-based FU are shown in a relative scale, so that the environmental behaviour of the four productions can be compared. Raspberry orchard was the most impacting system in 13 of the 18 studied categories, whereas blueberry orchard was the less harmful in most categories. It is noteworthy that contrary to what might be expected, the land productivity of raspberry (8333 kg/ha year) was higher than the land productivity of blueberry (6160 kg/ha year). The comparison results were mainly determined by the differences in the specific electric consumptions (15 kW h and 0.23 kW h per kg of raspberry and blueberry, respectively). Blueberry crop had the highest impact in WC category due to its high hydric requirements (0.16 m^3^/kg of fruit), whereas cape gooseberry showed the lowest impact in this category. On the contrary, this last orchard showed the worst performance in the LCU, SOD and ME categories due to the low land productivity and the high consumption of fertilisers together with the derived emissions.

#### Area-based FU

It has been reported that the choice of FU can be very important when comparing systems with different levels of productivity per ha, such as organic and conventional crops. Basset-Mens and Van Der Werf (2005), among others, have recommended testing multiple functional units in these cases. For example, Fotia et al. (2021) described that irrigated olive crops had a lower footprint per 1 ton than rained systems but showed higher impacts per cultivated area. Ronga et al. (2019) also reported different results for tomato crops depending on FU selected, i.e. when 1 kg of tomato was used as FU, organic systems had lower impacts than conventional ones, whereas results turned around when the analysis was carried out per 1 ha.

**Fig. 2:**
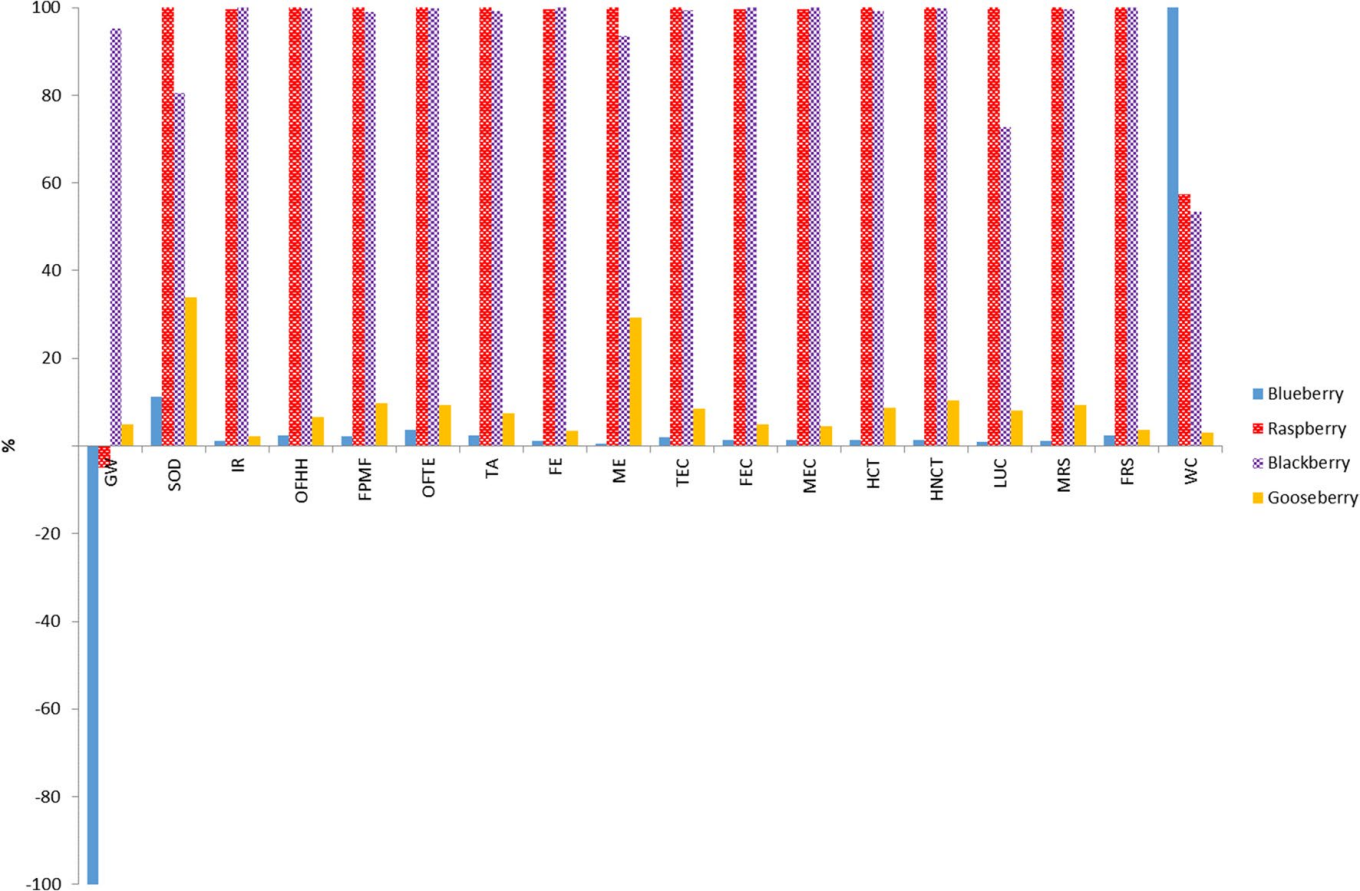
Comparison of characterization results obtained for different systems using Recipe Midpoint (H) method (FU: 1 ha)

As the berry crops here considered had different productivities, the comparison has been also carried out using an area-based FU. As can be observed in Fig. 2, the raspberry crop showed again the worst environmental performance, with the highest impact values in 16 of the 18 categories, closely followed by the blackberry crop that exhibited the highest impacts in 10 categories (both crops had very similar results for nine categories). Raspberry and blackberry orchards were located in a very small farm. Then, it must be taken into account that, in crops, economies of scale are usually observed with respect to energy efficiency, so that specific energy consumptions are usually higher for smaller farms. In fact, raspberry and blackberry crops had the highest electric consumption per area unit (around 13 kW h/m^2^ year) and also the highest fuel consumption (around 0.3 L of gasoline per m^2^). In addition, it was observed that the amount of fertilizers used per area unit was also quite higher for raspberry and blackberry (around 0.4 kg/m^2^ year), than for blueberry and cape gooseberry (below 0.05 kg/m^2^ year). Blueberry showed again the best environmental behaviour, except for WC category, which is in agreement with the fact that it is the crop that consumed less electricity per area unit (0.14 kW h/m^2^ year).

#### Price-based FU

**Fig. 3:**
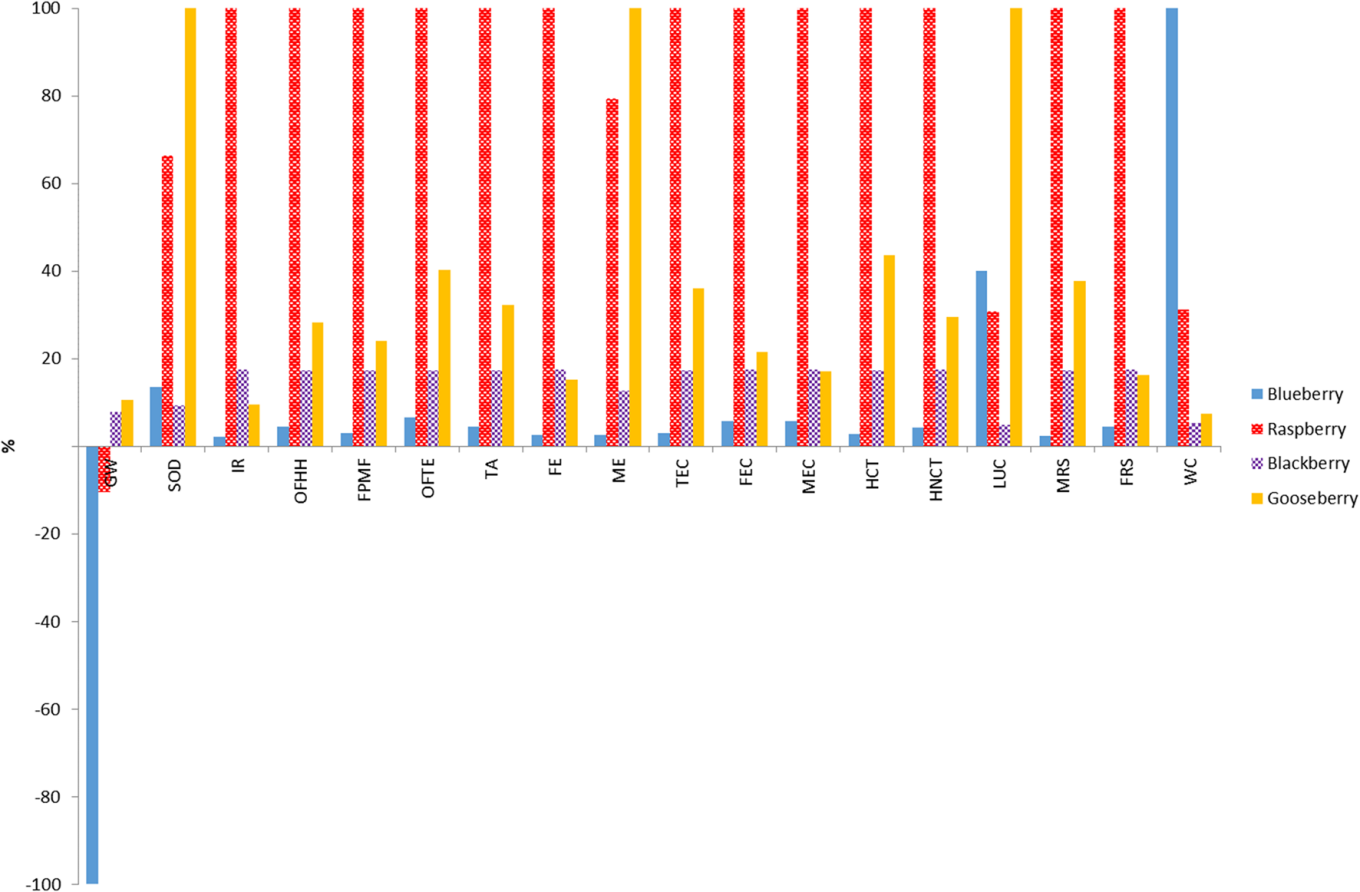
Comparison of characterization results obtained for different systems using Recipe Midpoint (H) method (FU: 1 euro)

In Fig. 3, results of LCA when the considered FU is the amount of each fruit that is sold at farm gate by 1 € are compared. There are big differences between the farm-gate prices of the different berries. As explained in “Materials and methods”, the considered prices ranged between 4 and 8 € following, from the cheapest to the most expensive, the next order: blueberry, raspberry, cape gooseberry and blackberry.

Qualitatively, results obtained in this case were very similar to that obtained with the mass-based FU. Therefore, again, raspberry orchard showed the highest impact in 13 categories, and blueberry orchard showed the lowest impact in 15 categories. However, a more detailed analysis showed that in the comparative study, the magnitude of the impacts for the most expensive berries, i.e. cape gooseberry and blackberry, showed a relative reduction in all categories, so that in some categories, the order in the impacts of some berries was reversed. For example, with the price-based FU, the worst behaviour in the GW category was shown by the cape gooseberry, whereas with the mass-based FU, it was shown by the blackberry crop. Likewise, in the SOD category, the blackberry showed the best environmental behaviour when the price-based FU was used, whereas, with the mass-based FU, the blueberry was the best in this category.

In line with this study, Da Silva et al. (2014) explored the use of the economic value at the farm gate as an alternative FU, comparing the impacts of chicken production in extensive and intensive systems on France and Brazil. This author determined that using mass-based FU was more relevant for production chain, but from consumers’ viewpoint, economic-based FU was more important. Sills et al. (2020) considered that an analysis based on prices could be inappropriate for a developing technology or making structural decisions due to the market volatility (consumer trends, inflation…).

#### Nutritional-based FU

Several proposals have been published to define a nutrient density index that can be used as complementary FU in LCA studies with the objective of expressing the environmental impact of foods in relation with their nutritional quality (Bianchi et al. 2020). As previously commented, FU selection strongly influenced the identification of sustainable foods (Masset et al. 2015). For example, fruits and vegetables have very low GHG emissions per 100 g of food but similar to meats and mixed dishes per 100 kcal. This is explained by the low energy density and high water content of fruits and vegetables in comparison with meat and fish. In addition, Sonesson et al. (2019) described a methodology that includes nutritional aspects in a FU to promote dietary changes and found, for example, that fruits and vegetables had lower impacts per nutritional unit than bread. Nutrient density indexes were shown to be useful for comparing foods between and within food groups (Bianchi et al. 2020). In this work, it has been used the concept of nutritional density unit (NDU) proposed by Dooren (2016) to compare the berry crops. The number of NDU per kg of fruit, calculated for each berry as explained in “Materials and methods”, ranged between 16 and 43 NDU/kg so that the berries follow, from the less to the most nutritive, the next order: blueberry, cape gooseberry, raspberry and blackberry, being the two last very similar.

**Fig. 4:**
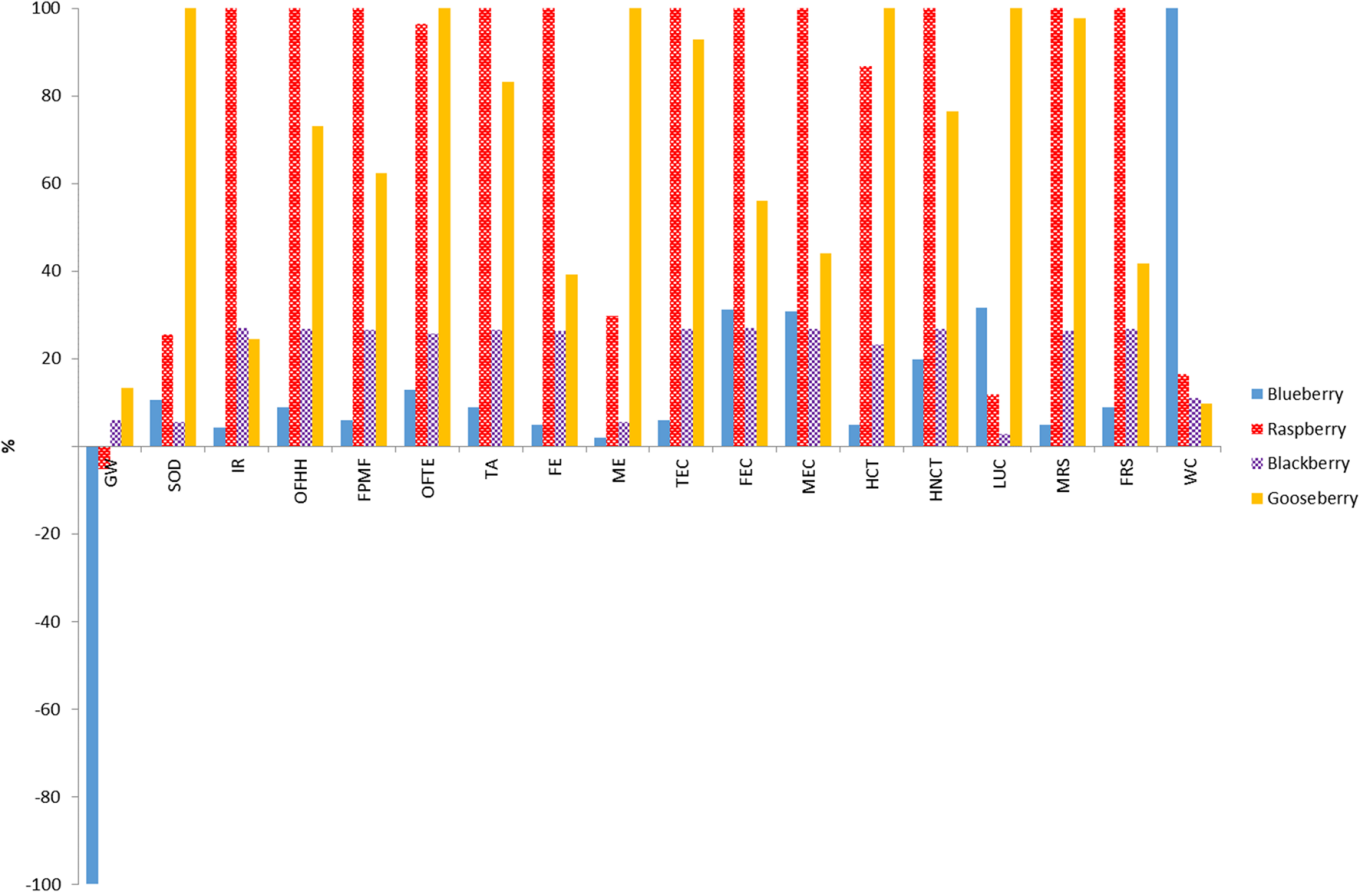
Comparison of characterization results obtained for different systems using Recipe Midpoint (H) method (FU: 1 NDU)

Figure 4 shows comparative LCA results when 1 NDU was used as FU. Again, qualitative results are similar to those obtained with the mass-based UF, i.e. raspberry exhibited the highest harmful impacts in 11 categories, and the least impacting crop was the blueberry in 13 categories. However, in this case, there was a change in the order of the blackberry and cape gooseberry crops. So, when the mass-based FU was used, blackberry was the second more impacting crop in most categories, whereas with the nutritional-based FU, this second position corresponded to the cape gooseberry crop. These changes were due to the fact that, to ingest the same amount of NDU, it is necessary to eat approximately the double of cape gooseberry than blackberry.

As above commented, although general results are similar, depending on the kind of FU considered, certain differences can be observed in the comparative analysis of the four berry crops. The use of a mass-based functional unit is very related with the productivity of the crop (Charles et al. 2006; Brentrup 2003). In contrast, the area-based functional unit should be used to consider the environmental impact on a local area (de Backer et al. 2009). If the goal of the study is to inform consumers, selecting a mass-, price- or nutritional-based FU might be more relevant than using an area-based-FU. This is in agreement with Jungbluth et al. (2000) who stated that if consumers could take into account additional product characteristics with respect to the associated environmental impacts, they could adapt their consumption habits to buy the most environmentally friendly.

### Carbon footprint of berries production systems

Some studies have demonstrated that European consumers are interested and concerned about climate change and, therefore, more likely to buy food products with CO_2_-labels (Feucht and Zander 2017). In this work, CF values have been obtained for the considered berries crops using different functional units. Based on ISO 14062, CF values have been calculated considering only fossil and biogenic CO_2-_eq emissions. CO_2_ uptake by plants, which was included in the GW category (Figs. 1, 2, 3 and 4), has not been taken into account for CF calculation.

**Fig. 5:**
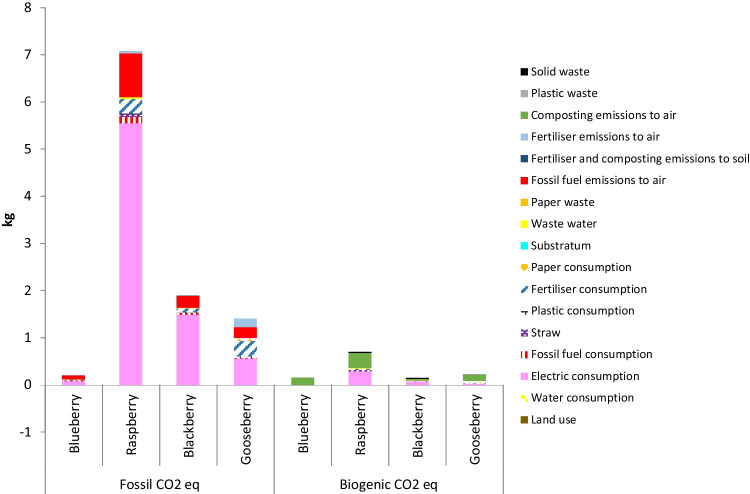
Comparison of carbon footprint obtained for the different systems using Greenhouse Gas Protocol (FU: 1 kg fruit)

**Fig. 6:**
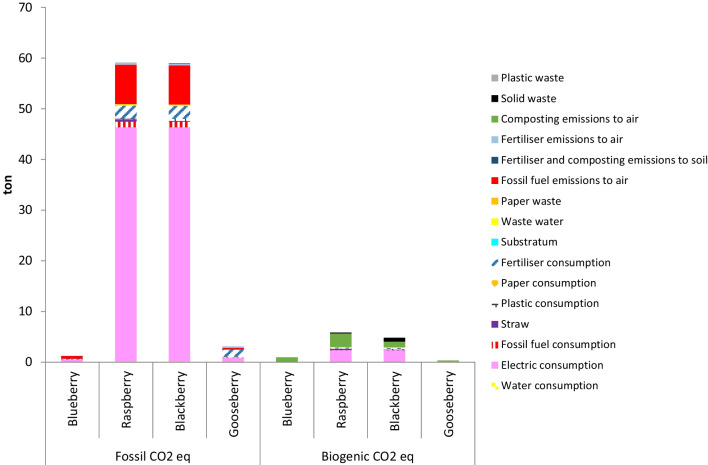
Comparison of carbon footprint obtained for the different systems using Greenhouse Gas Protocol (FU: 1 ha)

**Fig. 7:**
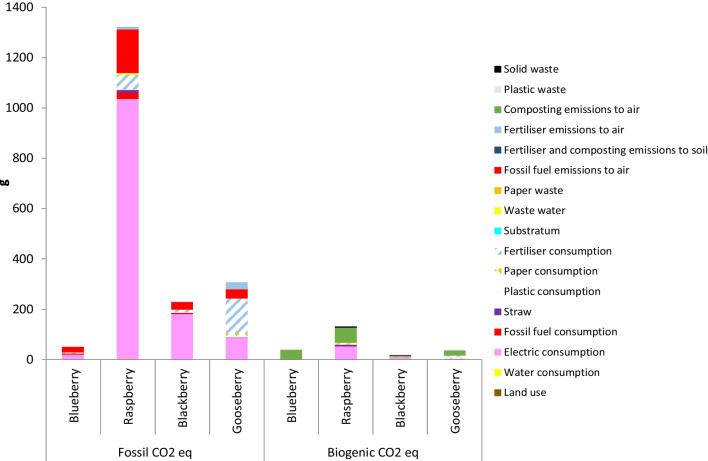
Comparison of carbon footprint obtained for the different systems using Greenhouse Gas Protocol (FU: 1 euro)

**Fig. 8:**
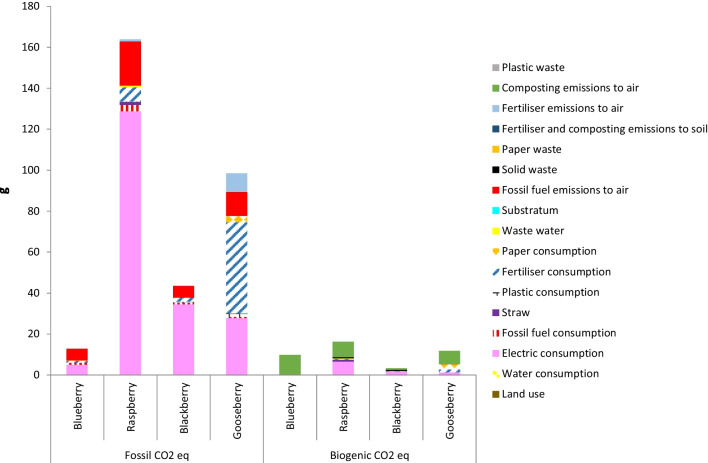
Comparison of carbon footprint obtained for the different systems using Greenhouse Gas Protocol (FU: 1 NDU)

As can be observed in Fig. 5, 6, 7 and 8, in all orchards, four subsystems, namely electric consumption, fertiliser consumption, fossil fuel emissions and composting emissions to air, were the main contributions to the total GHG emissions responsible for the calculated CFs. These results are in accordance to those reported by several authors, who found that GHGs derived from vegetables and fruit production were mainly originated by the consumption of fertilisers, energy and fuels (Cerutti et al. 2014; Girgenti et al. 2013; Ingrao et al. 2015; Keyes et al. 2015; Maraseni et al. 2010; Meier et al. 2015; Nikkhah et al. 2016). In the current work, fossil fuel emissions to air were mainly originated by the employment of gasoline or diesel for the use of machinery and transportation of materials within the crop system.

#### Mass-based FU

When the mass-based FU was used (Fig. 5), the order of the berries from the lowest CF to the highest was as follows: blueberry, cape gooseberry, blackberry and raspberry. The main contribution was in all cases the electric consumption (fruit storage refrigerators, lighting, irrigation system), followed by the emissions to air derived from the use of fossil fuel and the production of fertilisers.

The CF here obtained for blueberry was 0.37 kg CO_2_eq. per kg of fruit, value within the range reported in literature for this berry. In fact, most published CFs were between 0.20 and 0.80 kg CO_2_eq. per kg of blueberry (Cordes et al. 2016; Schein 2012), although in some cases, values above 1 kg CO_2_eq. per kg have been obtained (Rebolledo-Leiva et al. 2017; Pérez et al. 2022).

With respect to the cape gooseberry, the CF here obtained was higher than the blueberry CF, with a value of 1.63 kg CO_2_eq. per kg of fruit. Different authors (Frohmann et al. 2015; Graefe et al. 2013; Perez 2012) have carried out diverse studies on calculating the CF of cape gooseberry production in various regions of Colombia. In Novacampo, a value of 5.20 kg CO_2_eq per kg of fruit was obtained, being consumption of fertilisers and packaging the most impacting subsystems. In Ocati, this value increased to 6 kg CO_2_eq per kg in convectional orchards, mainly due to transportation. For Colombian organic crops (Frohmann et al. 2015; Graefe et al. 2013; Perez 2012), CFs between 4.76 and 7.11 kg CO_2_eq per kg have been reported, values considerably higher than the CF obtained in this work for the Spanish organic gooseberry crop. This is even more noteworthy if we take into account that the yearly productivity in the cape gooseberry crop here analysed was 1600 kg/ha, much lower than productivities above 10,000 kg/ha reported in Colombia.

Higher values of CF were here obtained for raspberry and blackberry orchards, i.e. 7.78 and 2.07 kg CO_2-_eq/kg respectively. On the contrary, Clune et al. (2017) obtained a CF of only 0.84 kg CO_2_eq per kg of raspberry. In Piedmont (Italy), the same values of CF were reported for raspberry and blackberry, specifically, 0.42 kg CO_2_eq/kg (Peano et al. 2015). The main harmful impacts in the mentioned study were originated by consumption of plastics on nursery and mulching, consumption of fertilisers and the irrigation system. In a similar way, studies carried out in Colombia and Chile found values between 0.18 and 2.40 kg CO_2_eq per kg of raspberry produced (Graefe et al. 2013; INIA 2009). As can be observed, the CF values published for raspberry and blackberry are quite lower than those estimated in this work.

#### Area-based FU

CFs have been also estimated using the area-based FU, with the aim of comparing the different effects on global warming derived from the different intensive cultivations from a cultivated area perspective. High differences can be observed among berries in Fig. 6. So, blueberry and cape gooseberry crops presented the lowest CF, i.e. 2.27 and 3.55 tons of CO_2_eq per ha and year, respectively, whereas the values obtained for raspberry and blackberry crops were much higher, i.e. 64.93 and 63.84 tons of CO_2_eq per ha and year, respectively. When literature CFs given per ha of different fruits and vegetables crops are compared, a wide range of values is observed, from a quarter of a ton to several tons of CO_2_eq per ha and year, depending on the kind of crop and the farming practises (Heusala et al. 2020; Knudsen et al. 2014; Ronga et al. 2019). For example, Proietti et al. (2014) found that the annual average GWP of the olive grove was 1.51 tons of CO_2_eq per ha and year. This author considered that the greatest impacts were originated by the use of fertilisers and pesticides, whereas in the present work, the main contribution was the electric consumption. In addition, it has been reported an average value of 7.44 tons of CO_2_eq per ha and year for the grapefruit sector in Spain (AILIMPO 2022).

#### Price-based FU

If GHG emissions are analysed using price-based FU (Fig. 7) for blueberry, raspberry, blackberry and cape gooseberry, the following values are obtained: 0.09, 1.45, 0.25 and 0.34 kg of CO_2_eq per euro, respectively. Again, blueberry presented the lowest value despite to the fact that it had the lowest price at the farm gate. However, in this case, blackberry CF is lower than cape gooseberry due to its higher price. Raspberry has the highest CF as occurred with the mass- and area-based FUs.

#### Nutritional-based FU

Dooren (2016) designed a FU that considered the nutrient density of foods; this unit reflects the food’s ‘function’ of supplying the essential macronutrients according to human energy needs. This author reported GHG emissions for berries ranging from 0.007 kg of CO_2_eq per NDU for red berry to 0.257 kg for strawberry. Values obtained in the present work (Fig. 8) were within the range reported, i.e. 0.023, 0.180, 0.047 and 0.110 kg of CO_2_eq per NDU for blueberry, raspberry, blackberry and cape gooseberry, respectively. The order followed by the considered berries with respect to the GHG emissions was the same as using the price-based FU. As the berries have relatively high nutritional properties and low associated GHG emissions, CFs given per NDU here estimated are lower than those reported for other food groups as grains, potatoes, oils, fats and cheese with emissions above 0.3 kg per NDU.

Independently of the FU, the lowest CF was obtained by the blueberry crop and the highest CF by the raspberry crop. However, the order of the blackberry and cape gooseberry crops was different depending on the selected FU. So, cape gooseberry presented lower CF than blackberry with the mass- and area-based FUs, whereas blackberry presented lower CF than cape gooseberry with the price- and nutritional-based FUs. In addition, with the area-based FU, blackberry CF is quite close to raspberry CF, whereas, with the other FUs, blackberry CF is much lower than raspberry CF. These results reveal the importance of selecting an appropriate FU according with the objectives pursued with the analysis.

## Conclusions

When environmental impacts from agricultural productions are analysed by LCA methodology, defining an accurate functional unit is crucial. According to ISO 14040 and 14,044, it is essential that FU aligns with the goal of the study, which determines how to interpret the results. The present work evidences the importance of an appropriate choice of FU when comparing the organic production of different berries, i.e. blueberry, raspberry, blackberry and cape gooseberry, in small orchards located in North Spain. Four functional units, namely, mass-, nutritional-, price- and area-based FU, have been considered with the aim to evaluate, respectively, four possible functions of the crops, i.e., to produce berries, to feed humans, to earn money and to cultivate land. Firstly, it is remarkable that the main contributions to the environmental impacts in all crops were related with the consumption of electricity and fertilisers. When the crops are compared, it was observed that independently of the selected FU, the blueberry and raspberry crops had the better and the worst performance respectively. However, some differences in the order of the crops emerged depending on the considered environmental category and the FU employed. For example, taking as criterion the total amount of GHG emissions produced by each farm studied, the order from the lowest to the highest CF varied with the FU. Hence, if the function was to obtain 1 kg of a fruit or to cultivate 1 ha of land, the order, from the less to the most environmental impacting system, would be blueberry, cape gooseberry, blackberry and raspberry. In contrast, if the function was to obtain an amount of fruit which can be sold by 1 € or which is equivalent to 1 NDU, the order would be blueberry, blackberry, cape gooseberry and raspberry. Therefore, it is clear that to compare crops producing different agricultural products, a misrepresentative choice of FU in relation with the function considered for the systems could lead to incorrect conclusions. In addition, when several functions are considered for the agricultural systems at the same time, the use of multiple functional units provides a global picture of their environmental behaviours, which can help to take more sensible decisions.

### Supplementary Information

Below is the link to the electronic supplementary material.

Supplementary file1 (DOC 71 KB)

## Data Availability

The data that support the findings of this study are available from the corresponding author upon reasonable request.
