# Supplementary material for: Evidencing the importance of the functional unit in comparative life cycle assessment of organic berry crops
Source: Environ Sci Pollut Res Int. 2024 Feb 24;31(14):22055–72. doi: 10.1007/s11356-024-32540-6 (PMC10948583; doi:10.1007/s11356-024-32540-6)
Supplement: Supplementary file 1 — Supplementary file1 (DOC 71 KB) [file 11356_2024_32540_MOESM1_ESM.doc]

**Evidencing the importance of the functional unit in comparative life cycle assessment of organic berry crops**

Reina Pérez, Fernando Argüelles, Amanda Laca, Adriana Laca*

Department of Chemical and Environmental Engineering, University of Oviedo

C/ Julián Clavería s/n, 33006, Oviedo, Asturias, Spain

*[lacaadriana@uniovi.es](mailto:lacaadriana@uniovi.es)

*Fig. S1 Contribution analysis based on the characterization results obtained using Recipe Midpoint (H) method (FU: 1kg blueberry).*

*Fig. S2 Contribution analysis based on the characterization results obtained using Recipe Midpoint (H) method (FU: 1kg raspberry).*

*Fig. S3 Contribution analysis based on the characterization results obtained using Recipe Midpoint (H) method (FU: 1kg blackberry).*

*Fig. S4 Contribution analysis based on the characterization results obtained using Recipe Midpoint (H) method (FU: 1kg gooseberry).*
